# Construction of a highly flexible and comprehensive gene collection representing the ORFeome of the human pathogen *Chlamydia pneumoniae*

**DOI:** 10.1186/1471-2164-13-632

**Published:** 2012-11-16

**Authors:** Christina J Maier, Richard H Maier, Dezso Peter Virok, Matthias Maass, Helmut Hintner, Johann W Bauer, Kamil Önder

**Affiliations:** 1 Department of Dermatology, Paracelsus Medical University, Salzburg, Austria; 2 Institute of Clinical Microbiology, University of Szeged, Szeged, Hungary; 3 Department of Microbiology, Paracelsus Medical University, Salzburg, Austria

**Keywords:** ORFeome, Chlamydia pneumoniae, Omics, Pathogen, Systems biology, Infectious diseases

## Abstract

**Background:**

The Gram-negative bacterium *Chlamydia pneumoniae* (Cpn) is the leading intracellular human pathogen responsible for respiratory infections such as pneumonia and bronchitis. Basic and applied research in pathogen biology, especially the elaboration of new mechanism-based anti-pathogen strategies, target discovery and drug development, rely heavily on the availability of the entire set of pathogen open reading frames, the ORFeome. The ORFeome of Cpn will enable genome- and proteome-wide systematic analysis of Cpn, which will improve our understanding of the molecular networks and mechanisms underlying and governing its pathogenesis.

**Results:**

Here we report the construction of a comprehensive gene collection covering 98.5% of the 1052 predicted and verified ORFs of Cpn (*Chlamydia pneumoniae* strain CWL029) in Gateway® ‘entry’ vectors. Based on genomic DNA isolated from the vascular chlamydial strain CV-6, we constructed an ORFeome library that contains 869 unique Gateway® entry clones (83% coverage) and an additional 168 PCR-verified ‘pooled’ entry clones, reaching an overall coverage of ~98.5% of the predicted CWL029 ORFs. The high quality of the ORFeome library was verified by PCR-gel electrophoresis and DNA sequencing, and its functionality was demonstrated by expressing panels of recombinant proteins in *Escherichia coli* and by genome-wide protein interaction analysis for a test set of three Cpn virulence factors in a yeast 2-hybrid system. The ORFeome is available in different configurations of resource stocks, PCR-products, purified plasmid DNA, and living cultures of *E. coli* harboring the desired entry clone or pooled entry clones. All resources are available in 96-well microtiterplates.

**Conclusion:**

This first ORFeome library for Cpn provides an essential new tool for this important pathogen. The high coverage of entry clones will enable a systems biology approach for Cpn or host–pathogen analysis. The high yield of recombinant proteins and the promising interactors for Cpn virulence factors described here demonstrate the possibilities for proteome-wide studies.

## Background

Bacteria are one of the most common causes of community acquired pneumonia. The Gram-negative bacterium *Chlamydia pneumoniae* (Cpn) is a prominent human pathogen responsible for respiratory infections like pneumoniae and bronchitis [1,2]. Moreover, the involvement of Cpn in other human diseases such as atherosclerosis [3], reactive arthritis [4], and myocarditis [5], or the association between Cpn infection and lung cancer [6], makes this pathogen an important risk factor in human disease. Furthermore, the bacterium’s obligate intracellular lifecycle can facilitate establishment of chronic persistent infections and/or reinfections following primary infection, thus posing a risk for chronic inflammatory disease [7,8].

About half of the 1052 open reading frames (ORFs) of Cpn encode proteins without any known function [9]. A flexible gene collection that contains all Cpn genes is a precondition to identify new or additional protein functions, e.g. through the discovery of possible interaction partners or the production of recombinant proteins for enzymatic assays or to test their influence on gene expression.

In recent years, numerous comprehensive gene collections termed ORFeomes have been constructed for organisms ranging from bacteria [10] to human [11]. The use of recombinational cloning, or Gateway® cloning [12,13], as a cloning strategy lends itself to diverse applications, from whole-genome sequencing to downstream ‘omics’ approaches. Cloning methods using traditional restriction endonucleases are too cumbersome and inefficient when hundreds of genes have to be cloned and analyzed further. Recombinational cloning enables high-throughput and large-scale ‘omics’ applications [14] with a wide choice of vectors, including Yeast Two-Hybrid (Y2H) vectors [15], Mammalian Two-Hybrid vectors [16], and ORF expression vectors. The availability of an ORFeome should accelerate systematic and organism-wide molecular research, improving our understanding of complex molecular networks governing virulence factors and pathogenesis.

Here we report the construction of a comprehensive gene collection covering 98.5% of the 1052 predicted and verified ORFs of Cpn (*Chlamydia pneumonia* strain CWL029) in Gateway® ‘entry’ vectors. This platform is highly flexible because the genes can easily be shuttled into any type of ‘destination’ vector to create an expression library for genome-wide studies such as Y2H mapping of novel Cpn protein–protein interactions (PPIs). Also, new target genes involved in host interactions can be identified. Array-based methods are already established to screen hundreds of ORFs for possible Cpn–human PPIs [17]. We verified the quality of the created platform by PCR amplification and DNA sequencing of entry clones. To demonstrate its functionality we performed a genome-wide Y2H analysis with three different Cpn genes: the EUO gene is a transcriptional regulator, and the flhA and fliF genes encode orthologs of flagellar proteins and are probable members of the Type-III secretion apparatus. Furthermore, to establish the suitability of the ORFeome for protein or proteome research, we demonstrated expression of panels of GST-tagged Cpn fusion proteins in *E. coli*. This newly generated, adaptable platform offers numerous advantages for high-throughput genomic and proteomic investigations into the molecular mechanisms and pathways involved in Cpn infection.

## Results

**Figure 1:**
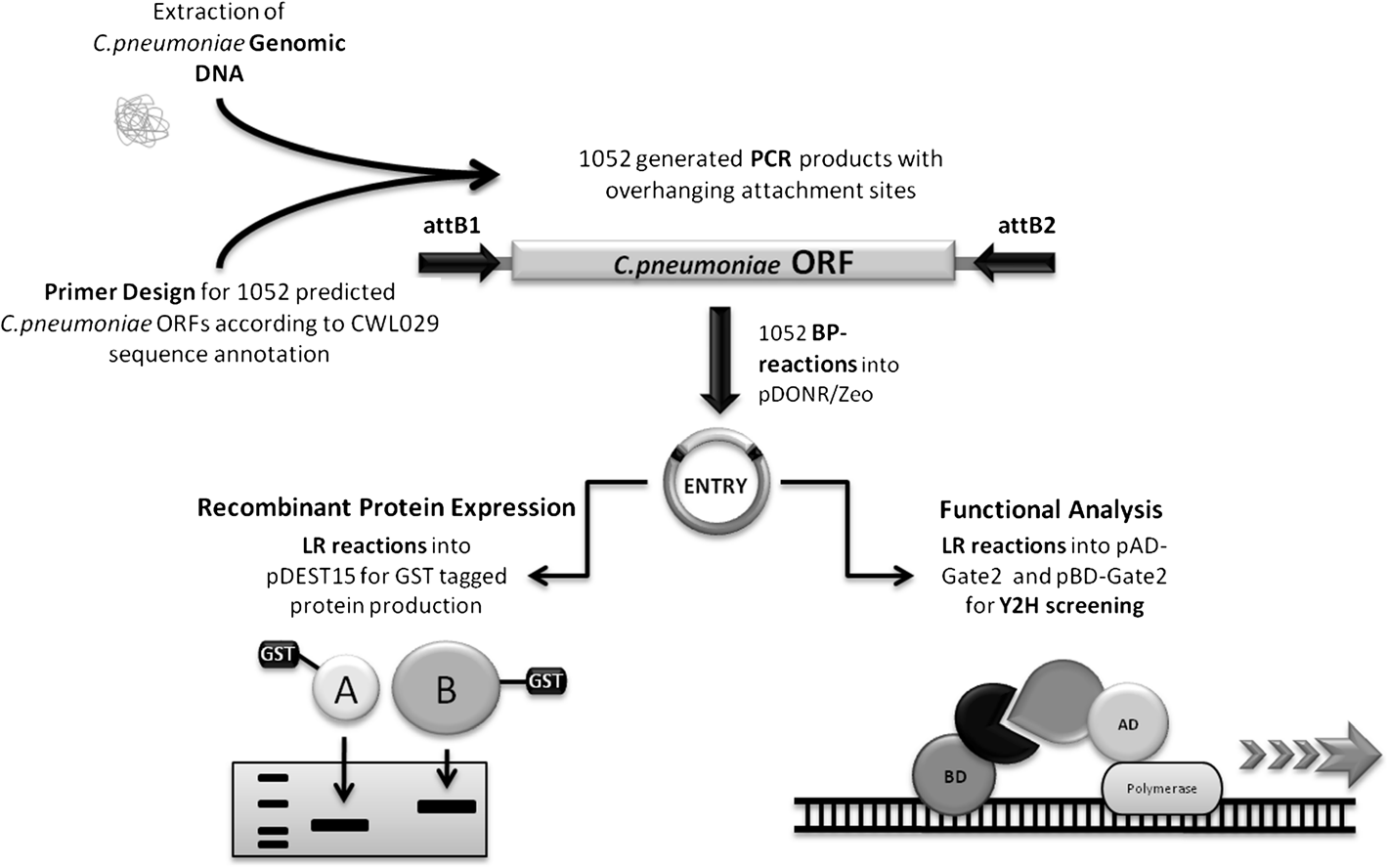
**Overview of the construction of the *C. pneumoniae *ORFeome.** The PCR products of the amplified ORFs were used in the first step of a BP reaction to create an Entry library, followed by PCR and sequencing for technical confirmation. For expression of recombinant proteins, the Entry library was used for LR reactions. Additionally, an Y2H library was generated for validation by functional analysis.

The overall strategy we employed for construction and characterization of the ORFeome of *C. pneumoniae* is summarized in Figure 1.

### The ORFeome of *C. pneumoniae* represents 98.5% of the predicted ORFs

For construction and characterization of the Cpn ORFeome, we employed the same strategy as we used in developing the first *Staphylococcus aureus* ORFeome [10]. For amplification of each Cpn ORF, the primers were designed to generate full-length attB1 and attB2 sites. To allow for the production of C-terminal fusion proteins, which can be expressed in appropriate destination vectors, the stop codons of each ORF were omitted in the reverse primers. Shine-Dalgarno (5^′^-GAAGGAGATA-3^′^) and Kozak (5^′^-ACCATG-3^′^) consensus sequences were incorporated into the forward primers such that the ATG in the Kozak sequence is in frame with the attB1 site, thus allowing N-terminal fusion proteins to be produced from destination vectors that contain N-terminal tags. After preparation of all Cpn PCR products for Gateway® cloning, a set of 300 arbitrarily chosen samples were analyzed by gel electrophoresis. All samples, apart from two exceptions, showed a DNA band of the expected size.

The PCR products were inserted into the Gateway®-compatible vector pDONR™/Zeo (Invitrogen, Carlsbad, CA, USA) by BP-cloning. The products resulting from site-specific recombination were transformed into *E. coli*. A portion of the cells was plated on solid medium containing Zeocin™, and the remainder was used to inoculate liquid medium containing Zeocin™ to generate bacterial stocks of pooled entry clones for long-term freezer storage. Up to five single colonies from each plate were tested in a colony-PCR with pDONR™/Zeo-specific primers. The resulting PCR products were detected by gel electrophoresis. Transformants which had an insert of the expected size were picked and grown in liquid medium containing Zeocin™ to generate bacterial glycerol stocks for long-term freezer storage. By this approach we obtained 869 transformants carrying the correct insert, which represents 83% of the 1052 predicted ORFs. Using both the positive single-colony glycerol stocks to inoculate liquid medium containing Zeocin™ and the liquid glycerol stocks for those ORFs for which no positive single-colony isolate was obtained, cultures were grown in 96-well plates and plasmid DNAs were prepared and used for PCR. For an additional 168 samples, an insert of the size expected for the ORF was observed. In total, we found 869 single entry clones and 168 ‘pooled’ entry clones, giving a resource of 1,037 entry clones (98.5% of the predicted ORFs). The remaining 1.5% yielded a PCR product of ~300 bp, derived from empty pDONR™/Zeo vector. As reported by Brandner et al. [10], we expect there to be additional positive single clones in the liquid glycerol stocks of the pooled entry clones. The single-colony glycerol stocks, the glycerol stocks of the pooled transformants, and the purified plasmid clones together constitute the ORFeome of *Chlamydia pneumoniae*.

### The identity of all single entry clones was validated by DNA sequencing

To verify that the inserted ORFs are in the correct reading frame and indeed correspond to the assigned identity, we took all plasmid preparations of the positive single colonies and subjected them to sequencing with a pDONR™/Zeo-specific forward primer. Ninety percent of the positive single clones were found to be correctly inserted, and a BLAST search of each sequence against the Cpn CWL029 genome (NCBI Refseq: NC_000922.1) confirmed the identity of each ORF. Almost all of the remaining 10% of positive single clones were found to have mutations in the binding region of the gene-specific forward primer, resulting in frameshift or missense mutations. Despite the presence of these mutations in the primer regions, the identity of these clones was confirmed by BLAST searches.

### Proteome-wide recombinant gene expression is possible by using the Cpn ORFeome

**Figure 2:**
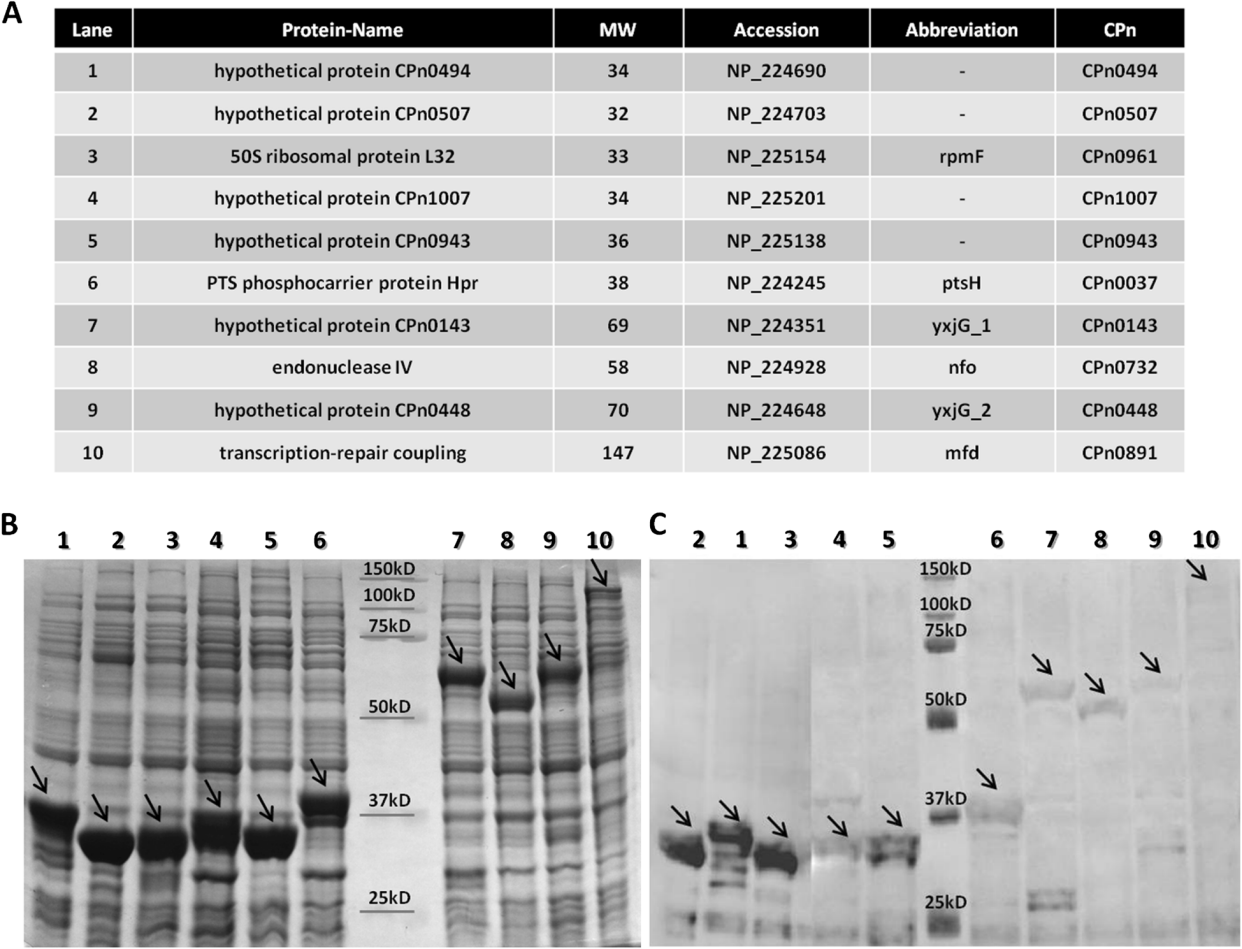
**Recombinant protein expression in *E. coli.*** Ten GST-tagged *C. pneumoniae* recombinant proteins (**A**) were loaded in equal amounts of total protein from crude lysates in each lane and subjected to SDS-PAGE and coomassie staining (**B**). Additionally the same crude lysates were examined by SDS-PAGE and western blotting (**C**). The given protein sizes are already calculated for GST-fusion proteins. The arrows indicate the recombinant proteins.

In order to examine all ORFs at the protein level we decided to shuttle randomly choosen single clones into the pDEST™15 vector (Invitrogen) designed to make a fusion protein with a GST-Tag. The resulting products were transformed into the *BL21*(DE3) protein expression strain of *E. coli*. Successful recombination of the choosen single clones was shown by PCR of single colonies obtained following transformation. For proteome-wide studies, the availability of recombinant proteins is the main bottleneck; therefore, we produced a panel of recombinant proteins directly from an arbitrarily chosen test set of 10 clones taken from the ORFeome cloned in pDEST™15 vector (Invitrogen). The clones were used to transform the *BL21*(DE3) protein expression strain of *E. coli*, and after induction of protein expression with IPTG, the cells were lysed and 2.5 μg total protein from the crude lysates were separated and analyzed on a protein gel with coomassie staining. As seen from the coomassie and western blot analysis in Figure 2, all 10 of the ORFs were highly expressed and a range of protein sizes from small to large was produced. In summary, robust expression of all 10 GST-tagged proteins demonstrates the functionality of the ORFeome.

### A proteome-wide yeast two-hybrid screen identifies several new interacting proteins for promising bait molecules

Proteins are the workhorses of the cellular machinery. They often function in huge protein complexes where the action of single proteins is mediated through their binding to other proteins in the complex. Moreover, there often exists cross-talk, mediated by interactions, between different protein complexes. Indeed, in all signal transduction pathways, the signal would become lost without functional PPIs [18]. Therefore, to understand a biological system, such as the relatively uncharacterized Cpn organism, it is essential to investigate novel protein interactions.

For that purpose and to demonstrate the new capabilities of the Cpn ORFeome, we performed three individual ORFeome-wide Y2H screens. As bait proteins, the Cpn genes flhA (Cpn0363; type III secretion system protein), fliF (Cpn0860; type III secretion system protein), and EUO (Cpn0561; hypothetical protein) were cloned in the Y2H pBD-Gate2 [15] vector. For all three bait molecules no autoactivation of reporter genes could be observed. A prey library was created by pooling all plasmid preparations of entry clones and transferring them by LR reaction into pAD-Gate2 [15]. Randomly chosen clones were selected for colony-PCR with pAD-Gate2-specific forward and reverse primers. The 20 clones showed different-sized PCR bands, from 400 bp up to 2.2kbp.

In all three screens, at least 1.7 x 10^5^ primary colonies, representing the same number of examined PPIs, were observed. On interaction-selective plates, we detected (and sequenced) two new interaction partners each for flhA and fliF. For the hypothetical protein EUO, four interactors were detected. These newly identified interaction partners are summarized in Table 1.

**Table 1:** Results of Y2H Analysis

**BAIT**	**Identified Interactor**	**Screen efficiency = tested binary PPI combinations**
type III secretion system protein **flhA** CPn0363	secretion chaperone **sycE** CPn0325	1.7*10^5^
type III secretion system protein **flhA** CPn0363	methyltransferase **ada** CPn0596	1.7*10^5^
type III secretion system protein **fliF** CPn0860	type III Secretion protein **fliN** CPn0704	3.6*10^5^
type III secretion system protein **fliF** CPn0860	hypothetical protein **CPn0693**	3.6*10^5^
**EUO** CPn0561	hypothetical protein **EUO** CPn0561	4.2*10^5^
**EUO** CPn0561	hypothetical protein **CPn0423**	4.2*10^5^
**EUO** CPn0561	hypothetical protein **CPn1007**	4.2*10^5^
**EUO** CPn0561	PTS phosphocarrier protein Hpr **ptsH** CPn0037	4.2*10^5^

## Discussion

In this work we described the construction of a comprehensive and flexible gene collection of *Chlamydia pneumoniae*. This ORFeome consists of 869 ORFs maintained as homogeneous plasmid DNAs purified from single colonies plus 168 ORFs purified from separate ‘pools’ of entry clones. This coverage of 98.5% of all predicted Cpn ORFs is similar to the coverage reported for other bacterial ORFeomes [10,19-21]. Such ORFeomes offer a flexible resource with which to undertake functional genomic studies, including high-throughput screens [22-24]. In our own laboratory we have successfully produced protein arrays/chips [17] whereon thousands of proteins can be tested against other proteins or peptides. The described Cpn ORFeome is the basis for functional high-throughput analysis of this organism, providing a flexible tool for quick and efficient genome-wide examination and discovery.

We assessed the quality of the ORFeome by PCR and agarose gel-electrophoresis of all the entry clones obtained. Sequencing all positive single clones confirmed the identity of the entry clones. To minimize the occurrence of PCR-induced mutations, we optimized the PCR conditions, used a proofreading-capable DNA polymerase, and kept the number of amplification cycles low. Functional validation was done by recombinant protein production and PPI analysis using the Y2H system. According to our previous work [10], we did not purify the first PCR products, because it does not minimize the possibility of primer dimers created by the secondary attachment primers. These primer dimers are the putative reason for the existence of samples for which no positive single clone could be found. In the present study we spot-tested 300 arbitrarily chosen first PCR products. Almost all products delivered a PCR band of the expected size. In addition, we examined PCR samples of negative candidates (no positive single clone) where the first-generated PCR product delivered, like the other samples examined, a strong PCR band. In most cases we could isolate positive candidates by investigating up to 20 clones of one reaction, affording a way to clone single genes but not hundreds of genes. We failed to produce PCR products for 15 ORFs. This could be due to possible errors in annotation of the CWL029 genome or, more likely, because of nucleotide differences between our Cpn isolate and the sequenced CWL029 genome or for technical reasons related to primer design and effectiveness specific to each missing ORF. The single entry clones are of higher quality than the pools of entry clones because they are homogeneous, and LR reactions performed subsequently with the pure clones will be more efficient. However, for reasons of economy and because of time constraints, it was not possible to screen every colony to obtain a pure isolate of the appropriate ORF-containing entry clone. However, we expect that additional positive single transformants are present in the liquid glycerol stocks. In future work, this version of the Cpn ORFeome may be extended by isolating more positive single clones.

Ninety-seven positive single clones (10% of the positive single clones) failed validation by DNA sequencing. Almost all of the affected genes harbor a mutation in the binding region of the primary forward primer, resulting in frameshift mutations. However, this type of problem could be solved by using primers of higher quality than the used Mass Spectrometry checked primers in this work. Another solution could be to re-order the 10% defective primers and re-clone the defective clones or to correct the mutations by using mutagenesis kits. Although these genes are not in frame with the commonly used Gateway® destination vectors, there is the possibility of transferring them to other vectors having the appropriate reading frame. Alternatively, there already exist reading-frame independent vectors suitable for Gateway® cloning [15,16]. The use of such vectors would increase the overall coverage of positive single clones.

The present work provides the first nearly complete ORFeome of Cpn. Production of recombinant proteins in the present system can be achieved with the well-known GST fusion tag. This tag could be used for further protein purification or affinity studies such as GST Pull-Down for validation of protein–protein interactions. We successfully produced fusion proteins of ten randomly chosen clones. ORFeome-wide protein chips already exist [25-27] and could be applied to our Cpn collection for analyzing not only protein–protein interactions, but also the ability of proteins to bind to small molecules as well as other proteins. A special field in the case of Cpn would be the use of proteome chips in drug and drug target discovery, because such chips can provide a variety of systems for detecting drug–protein interactions among all proteins. The technique for proteome chips already exists in our working group, and was verified by finding a small epitope to a known antigen, another type of protein–peptide interaction [17].

As a validation of our interaction screen, first we used FlhA, a Type-III secretion apparatus member. The Y2H screen identified the Type-III secretion chaperone sycE as an interaction partner. SycE is related to the class-I chaperones involved in binding of effector proteins during the secretion process. Since FlhA is located in the inner membrane and is involved in flagellar protein secretion, it is possible that SycE interacts with FlhA during the export of flagellar or other proteins. Supporting this finding, it was previously reported that another chaperon, FliJ, is also an interaction partner of FlhA [28]. We also identified the methyltransferase ada, which in *E. coli* is known to be involved in the repair of DNA damage induced by methylating agents. The link between ada and the Type-III secretion apparatus is unknown, but it is worth mentioning that chlamydia transports other methyltransferases to influence host gene expression via Type-III secretion [29].

The second tested bait protein, FliF, is a member of the MS ring of the Type-III injectisome [30]. FliF is a previously described binding partner of FlhA [31], but we could not detect this interaction. Although, the Y2H system is a powerful system to detect PPIs, nevertheless not all interactions can be detected within a single screen and further reasons could be loss of interaction due to the fusion with the yeast GAL4 protein domain, or the requirement that the interaction between bait and prey are obliged to happen in the nucleus of yeast cells. There are a couple of reasons leading to false-negatives in the Y2H system, a drawback actually existing in all PPI-methods. On the other hand, we identified FliN, a putative C-ring member [30], as an interaction partner of FliF.

Our third bait protein was EUO, a chlamydial protein expressed early in the developmental cycle. EUO is able to bind to AT-rich sequences [32] and also has been shown to have a histone H1-specific protease activity [33]. While the potential interaction partners of EUO are not known, we identified EUO itself, two hypothetical proteins, and the metabolic enzyme phosphocarrier protein ptsH as interacting partners. The exact roles of these interactions remain to be identified.

These screens were performed in a low-scale manner with a test Cpn protein set, but generation of an Cpn intrapathogen ‘interactome’ is conceivable through ORFeome-wide PPI screening by automated procedures. Likewise, a system-wide screen for host–pathogen PPIs could be conducted by screening against other available ORFeomes, e.g. the human ORFeome [11], which would produce a detailed description of the host–pathogen interface at the molecular level, leading to improved understanding of Cpn pathogenesis in humans.

## Conclusion

We achieved nearly complete cloning of the first version of the Cpn ORFeome, and furthermore demonstrated the feasibility of a respective expression ORFeome in the form of recombinant protein-producing vectors and pooled Y2H prey vectors. We showed that these collections are functional by recombinant protein production in *E. coli* and by a protein interaction assay. The availability of the Cpn ORFeome with appropriate Gateway®-compatible destination vectors offers many possibilities for proteome-wide studies, including systematic Y2H PPI screenings, and fabrication of protein chips or microarrays, which could enable antigen-profiling for vaccination strategies and so on. This first version of the Cpn ORFeome can be extended in future work by addition of the missing ORFs or inclusion of strain-specific variations.

## Methods

### Primer design

The DNA sequence of *C. pneumoniae* was obtained from the NCBI Genome Database (NCBI; Refseq: NC_000922.1). Primary gene-specific forward primers were designed by adding the sequence 5^′^-AAAAAGCAGGCTTGGAAGGAGATAGAACCATG-3^′^ to the 5^′^ end of the first 20 to 30 nucleotides of each ORF. Gene-specific reverse primers were constructed by adding the nucleotides 5^′^-GTACAAGAAAGCTGGGTA-3^′^ to the 5^′^ end of the last 20 to 30 nucleotides of the complementary strand of the ORF. The gene-specific parts of the primary forward and reverse primers were chosen to give similar annealing temperatures during the PCR, between 40–60°C. The 1052 primer pairs were obtained in a 96-well format.

### PCR amplification of the ORFs

As template DNA for PCR, Cpn genomic DNA was isolated using a QIAamp® DNA Mini Kit (Qiagen, Hilden, Germany) from the vascular chlamydial strain CV-6, which was recovered from chronicle vascular infection [34] and which has commonly been used in cell biological studies [35]. The 1052 PCRs were performed in 96-well plates containing 50-μl assay volumes consisting of 1.25 U Platinum Pfx polymerase (Invitrogen), 1 mM MgSO_4_, dNTP mix (0.3 mM each), primary forward and reverse primers (0.1 μM each), secondary adapter forward and reverse primers (0.4 μM each), 10× Pfx amplification buffer (5 μl) and *C. pneumoniae* genomic DNA (10 ng). The sequence of the secondary forward adapter primer is 5^′^-GGGGACAAGTTTGTACAAAAAAGCAGGCTTG-3^′^ and that of the secondary reverse adapter primer is 5^′^-GGGGACCACTTTGTACAAGAAAGCTGGGTA-3^′^. After the first few cycles, enough DNA template was produced by the primary forward and reverse primers to enable the secondary adapter primers to bind and extend the first PCR products to generate the full-length attB1 and attB2 sites flanking the ORFs. All 4 primers were used together in the same PCR. The 25 PCR cycles (94°C for 30 s, 48°C for 30 s, and 72°C for 1 min/kb) were preceded by heating to 94°C for 5 min and were followed by a 7-min incubation at 72°C. PCR products were used immediately without purification and then stored at −20°C.

### attB × attP recombination reactions - BP reactions

The Gateway®-compatible, amplified ORFs were recombined into the vector pDONR™/Zeo (Invitrogen) by using BP Clonase™ II Enzyme Mix (Invitrogen). In 96-well plates, samples containing 2 μl unpurified PCR product, 1 μl BP Clonase™ II Enzyme Mix, 150 ng pDONR™/Zeo plasmid and TE buffer, pH 8.0, up to 10 μl were incubated overnight at 25°C. After adding 1 μg proteinase K (Invitrogen) and incubating at 37°C for 30 min, the BP reactions were directly used for bacterial transformation. The reactions were stored at −20°C.

### Transformation

A 3-μl aliquot from each of the 1052 BP reactions was added to One Shot® TOP10 chemically competent *E. coli* (Invitrogen) using the manufacturer's protocol and 96-well plates. After heat-shock at 42°C for 30 s, 50 μl of SOC medium (Invitrogen) was added to the transformation reactions and the samples were incubated for 1 h at 37°C. After the incubation, 20 μl of each sample were plated onto low-salt LB solid medium containing 80 μg/ml Zeocin™ (Invitrogen) and incubated overnight at 37°C to produce single colonies. The remainder of the transformation reaction was used to inoculate 150 μl of low-salt LB liquid medium containing 80 μg/ml Zeocin™. These cultures were also incubated at 37°C overnight to generate transformants for long-term storage by adding glycerol and freezing at −80°C.

### Colony PCR of bacterial clones

A single colony from each transformation reaction was analyzed by PCR to verify the correct size of the inserted ORF. The 1052 PCRs were performed in 96-well plates containing 50-μl samples with 2.5 U BioTherm™ Polymerase (Genxpress, Wiener Neudorf, Austria), pDONR™/Zeo-specific forward primer (5^′^-GTAAAACGACGGCCAG-3^′^) and reverse primer (5^′^-CAGGAAACAGCTATGAC-3^′^) (0.3 μM each), dNTP mix (0.2 mM each) and 10× BioTherm™ reaction buffer (5 μl). Colonies were picked with a sterile pipette tip and transferred to the wells of the 96-well plate. The 40 PCR cycles (94°C for 30 s, 55°C for 30 s, and 72°C for 1 min/kb) were preceded by heating to 94°C for 5 min and followed by a 7-min incubation at 72°C. The sizes of the PCR products were determined by agarose gel electrophoresis and midori green staining. Entry clones with inserts having the expected size were inoculated in 150 μl low-salt LB liquid medium containing 80 μg/ml Zeocin™ and incubated overnight at 37°C to generate glycerol long-term frozen stocks. Up to five different single colonies from each transformation were screened by this PCR method till a positive single colony was found. In cases where the PCR failed to verify a positive colony, 'pooled' entry clones were analyzed. For that, an aliquot of the entire transformation reaction was cultured in liquid LB medium overnight at 37°C. The entire overnight culture was subjected to plasmid isolation and tested again by PCR. Plasmid preparations from cultures that were positive by PCR indicated the presence of correctly cloned ORFs; at worst, they consisted of a mixed population of cells having empty plasmids and ORF-containing plasmids.

### Glycerol long-term frozen stocks and plasmid preparation

Overnight cultures were mixed with glycerol to a final glycerol concentration of 40% for long-term storage at −80°C. Five milliliters of low-salt LB liquid medium containing 80 μg/ml Zeocin™ were inoculated with 2 μl of overnight bacterial culture. After overnight growth at 37°C, plasmids were purified with a GenElute™ Plasmid Miniprep Kit (Sigma-Aldrich, St. Louis, MO, USA), and sequenced with a pDONR™/Zeo-specific forward primer (5^′^- GTTTTCCCAGTCACGAC −3^′^).

### attL × attR recombination reactions - LR reactions

Entry vectors were set up in LR reactions to recombine the gene of interest into several destination vectors. The destination vectors used were pDEST™15 (Invitrogen), pBD-Gate 2, and pAD-Gate 2 [15]. The two Gate vectors are in-house-generated Gateway®-compatible pGADT7 and pGBKT7 plasmids of the Matchmaker™ GAL4 Two-Hybrid system (Clontech, Mountain View, CA, USA) for yeast two-hybrid analysis. Samples containing 2 μl of prepared entry clone, 1 μl LR Clonase™ II Enzyme Mix (Invitrogen), destination vector (150 ng), and TE buffer, pH 8.0, to 10 μl were incubated at 25°C overnight. After adding 1 μg proteinase K (Invitrogen) and incubating at 37°C for 30 min, the LR reactions were directly used for plasmid transformation into *E. coli*. The reactions were stored at −20°C. Bacteria were transformed with the LR reactions as described above using the appropriate antibiotic: 100 μg/ml ampicillin for pDEST™15 and pAD-Gate2; and 50 μg/ml kanamycin for pBD-Gate2. Colony PCRs were performed as described above to verify the successful cloning by using vector-specific primers. The expression clones were prepared using a GenElute™ Plasmid Miniprep Kit (Sigma-Aldrich).

### Recombinant protein production

Prepared pDEST™15 expression vectors harboring the desired ORFs were used to transform chemically competent *E. coli* BL21(DE3) (Invitrogen), and the cells were plated onto LB solid medium containing 100 μg/ml ampicillin. Single colonies from each plate were used to inoculate 5 ml LB liquid medium containing 100 μg/ml ampicillin. Cells were grown overnight at 37°C and protein expression was induced the next day by inoculating the whole overnight culture into 50 ml fresh medium containing 100 μg/ml ampicillin and IPTG (1 mM) and incubating for a further 4 h. The cells were lysed and protein expression was shown by SDS-PAGE and coomassie staining. The proteins were further analysed by standard western blot procedures. The primary antibody was the commercially available mouse anti-GST (B-14) (sc-138 B; Santa Cruz Biotechnology, Santa Cruz, CA). For detection, an HRP conjugated polyclonal rabbit anti-Mouse antibody (P0161; Dako, Glostrub, Denmark) was used.

### Genome-wide Y2H screening

As a functional test of our *C. pneumoniae* ORFeome library, we shuttled all entry clones into the Gateway®-compatible Y2H destination vector pAD-Gate2 to serve as a prey library in a screen for interactions with three proteins, two of them involved in Type III secretion (see below). To construct the library, all plasmid preparations of entry clones were pooled together and 2 μl of the pooled entry library were used in a standard LR reaction with pAD-Gate2. To construct the bait molecules, entry clones encoding the full-length genes flhA (CPn0363), fliF (CPn0860) and EUO (CPn0561) were shuttled into the GAL4 DNA-binding domain (BD)-containing vector pBD-Gate2 [15], and sequenced. In an additional experiment, in which the known interaction between p53 and SV40 large T antigen [36] served as a positive control, the constructs were found to have no capacity to non-specifically activate reporter genes. The prey library and the bait were used simultaneously to co-transform the haploid *Saccharomyces cerevisiae* strain AH109 (genotype: *MATa, trp1-901, leu2-3, 112, ura3-52, his3-200, gal4*Δ*, gal80*Δ*, LYS2:: GAL1*_*UAS*_*GAL1*_*TATA*_*-HIS3, GAL2*_*UAS*_*-GAL2*_*TATA*_*ADE2; URA3::MEL1*_*UAS*_*-MEL1*_*TATA*_*-lacZ MEL1*) according to a standard high-efficiency lithium acetate method, and finally plated onto (i) a nutritionally selective medium deficient in tryptophan and leucine (SD/-leu-trp) to test for positive plasmid uptake and general transformation efficiency, and (ii) SD/-trp-leu-ade-his synthetic medium to select for actual reporter activity. The plates were incubated for at least 4 days at 28°C. The prey library vectors from primary positive yeast candidate clones were recovered by plasmid isolation via digestion with 10 U lyticase (Sigma-Aldrich), addition of 10% SDS, one freeze-thaw cycle, and purification by using a Wizard® SV gel and PCR clean-up system (Promega, Madison, WI, USA). The plasmids were then amplified in *E. coli* DH5α cells and reintroduced back into the yeast strain AH109 together with the bait or empty pBD-Gate2 (as a control) and finally retested on SD/-trp-leu-ade-his in order to verify their reporter activity before the sequence of the clone was determined by using vector-specific primers. Bioinformatical analyses were conducted through the National Center for Biotechnology Information (NCBI) by using the basic local alignment search tool (BLAST) accessible via http://blast.ncbi.nlm.nih.gov/Blast.cgi, the ExPASy Proteomics Server ( http://www.expasy.ch/), the Universal Protein Resource UniProt ( http://www.uniprot.org/) and the SWISS-PROT knowledgebase ( http://expasy.org/sprot/).

## Competing interests

The authors declare no competing interests.

## Authors’ contributions

KÖ provided the original concept of the study, adjusted work related steps, supervised the study and contributed to writing the paper. CJM and RHM performed all of the work related steps, were responsible for data analysis, and prepared the manuscript. DPV and MM provided comments as well as scientific support and important revisions to the manuscript. JWB and HH gave scientific support to the study. All authors read and approved the manuscript.
